# Identification of Molecular Subtypes for Hepatocellular Carcinoma Based on Ubiquitin‐Conjugating Enzyme E2 (UBE2)‐Related Genes to Assess Prognosis and Immune Landscape

**DOI:** 10.1002/iid3.70409

**Published:** 2026-03-30

**Authors:** Shiqi Zhou, Chunwei Liu, Xiaodong Meng, Lingyu Kong, Wei Liu, Yuan Tian

**Affiliations:** ^1^ Department of Interventional Medicine North China University of Science and Technology Affiliated Hospital Tangshan Hebei China; ^2^ Department of General Surgery Kailuan General Hospital, Tangshan Hebei China; ^3^ Department of Minimally Invasive Hepatobiliary Surgery North China University of Science and Technology Affiliated Hospital Tangshan Hebei China; ^4^ Oncology Department of integrated Chinese and Western medicine North China University of Science and Technology Affiliated Hospital Tangshan Hebei China; ^5^ Affiliated Hospital of Tangshan Vocational & Technical College Tangshan Hebei China; ^6^ Department of Hepatobiliary and Pancreatic Surgery 1 North China University of Science and Technology Affiliated Hospital Tangshan Hebei China

**Keywords:** drug sensitivity, hepatocellular carcinoma, immune landscape, prognostic biomarkers, UBE2‐related genes

## Abstract

**Background:**

Hepatocellular carcinoma (HCC) is a prevalent and highly aggressive cancer, characterized by elevated morbidity and mortality. Ubiquitin‐conjugating enzyme E2 (UBE2) plays a crucial role in regulating HCC development, although the underlying mechanisms remain poorly understood.

**Methods:**

HCC patient transcriptomic and clinical datasets were sourced from The Cancer Genome Atlas database. Patients were classified into two distinct subtypes using the K‐means clustering method. Prognostic genes were identified through univariate and multivariate Cox regression, as well as least absolute shrinkage and selection operator regression. A nomogram was developed to predict patient prognosis, which was subsequently validated using the independent GEO dataset, GSE14520. Extensive model validation was performed to assess its prognostic significance. Immune landscape characterization was conducted using Single Sample Gene Set Enrichment Analysis (ssGSEA), ESTIMATE, and CIBERSORT algorithms. Drug sensitivity was also evaluated to identify potential therapeutic options.

**Results:**

Based on the expression profiles of 12 UBE2‐associated genes, we classified patients into two subtypes and identified six UBE2‐related genes as prognostic biomarkers. The risk score effectively predicted patient outcomes, with high‐risk individuals showing reduced survival and the low‐risk group characterized by elevated immune cell infiltration and unique immune checkpoint expression patterns. Additionally, potential drugs were identified, and drug sensitivity for HCC was evaluated.

**Conclusion:**

In this study, we established a prognostic risk model for HCC with strong predictive performance. Risk‐based stratification revealed its associations with immune infiltration, immunotherapy response, and drug sensitivity. These findings offer new insights into survival prediction and clinical features in patients with HCC.

## Summary

- Molecular subtypes were established using UBE2‐related genes, along with a corresponding prognostic model.
- This study identified six biomarkers that may serve as prognostic biomarkers for HCC: FTCD, LGALS3, PAGE1, RAMP1, RAMP3, and SLC1A7.
- Patients with a higher risk score exhibited immune escape and worse immunotherapy outcomes.

## 1 Introduction

Representing a major global health burden, liver cancer is the sixth most diagnosed cancer and the fourth most common cause of cancer death [1]. Primary liver cancers are mainly classified into hepatocellular carcinoma (HCC) and intrahepatic cholangiocarcinoma [2]. HCC is the predominant subtype, accounting for over 90% of primary liver cancer cases and representing the third leading cause of cancer‐related mortality globally [3, 4]. Chronic hepatitis B and hepatitis C virus infections, which drive persistent inflammation and cirrhosis, remain the major risk factors for HCC and contribute substantially to its global burden [5]. In addition, alcohol‐related liver disease and metabolic‐associated steatotic liver disease have emerged as important and increasingly prevalent contributors to HCC incidence [6]. Despite advances in treatment, the prognosis for HCC remains poor, with similar age‐standardized rates for incidence and mortality, presenting a global clinical challenge [7]. Given the variety of treatment options and the potential complications from liver dysfunction, a multidisciplinary team—including hepatologists, radiologists, surgeons, oncologists, and pathologists—is essential for determining the optimal approach for HCC. HCC management typically involves surgery, liver transplantation, locoregional interventions, and chemotherapy, with notable progress in systemic treatments [8], particularly with the rise of immunotherapy and targeted therapies [9, 10, 11]. Immune checkpoint (ICP) inhibitor therapy has shown potent antitumor activity in a subset of patients. The anti–PD‐L1 antibody atezolizumab combined with the VEGF‐neutralizing antibody bevacizumab has become, or is emerging as, a first‐line treatment for HCC, while anti–PD‐1 agents such as nivolumab and pembrolizumab are used in some regions as subsequent therapy following TKI treatment [12]. Other immunotherapeutic strategies, including adoptive T‐cell therapy, vaccines, or oncolytic viruses, have yet to demonstrate consistent clinical efficacy [13]. Due to the heterogeneity of HCC and the complexity of treatment options, there is an urgent need for reliable prognostic biomarkers to guide therapy and improve patient survival rates.

In eukaryotes, ubiquitin regulates the stability and activity of central proteins via the UPS, and aberrations in this system are associated with malignancies, neurodegeneration, and infections [14]. As a fundamental biochemical mechanism, ubiquitination governs multiple cellular processes, such as signaling pathways, cell death, protein turnover, and immune responses [15]. The ubiquitin‐conjugating enzyme E2 (UBE2) family is involved in ubiquitination, which plays an important cellular mechanism for the degradation of abnormal or short‐lived proteins [16]. More than 40 UBE2 enzymes have been characterized, and their aberrant activity influences cell cycle, apoptosis, DNA repair, and tumorigenic signaling pathways, making them promising candidates for cancer diagnosis, targeted therapy, and prognosis prediction [17]. HCC development is closely associated with chronic inflammation and dysregulated immune responses, which not only drive tumor progression but also affect therapeutic efficacy [18]. Understanding the molecular mechanisms underlying tumor‐immune interactions is therefore critical for improving prognosis and guiding treatment strategies. The ubiquitin‐proteasome system, particularly the UBE2 family, has been implicated in regulating key immune and inflammatory pathways in various cancers [19, 20], implying that genes related to UBE2 may represent promising biomarkers and targets for HCC therapy [21, 22]. The new studies have been shown that UBE2 is associated with various malignant tumors, such as cervical cancer [23], ovarian cancer [21], HCC, and so on [24]. However, although some UBE2 family members have been reported in HCC, comprehensive analyses of their expression patterns, molecular subtype characteristics, and associated immune landscapes remain lacking.

In this study, we leveraged comprehensive bioinformatics analyses to identify UBE2‐related genes, define novel prognostically relevant molecular subtypes, and characterize their biological functions as well as their potential utility in predicting patient outcomes and guiding therapeutic strategies in HCC. These analyses were performed using the Cancer Genome Atlas (TCGA) HCC samples covering patients across all clinical stages (stage I–IV).

## 2 Methods

### 2.1 Date Collection

RNA sequencing data and clinical information from 415 samples (50 normal and 365 tumor) were obtained from the TCGA database. Patients from the TCGA cohort were randomly assigned to training and internal validation groups at a 7:3 ratio. For independent validation, the GSE14520 dataset, comprising 85 tumor and 136 normal samples, was obtained from the Gene Expression Omnibus (GEO) database. A set of UBE2‐associated genes was obtained from the GeneCards database using the keyword “Ubiquitin Conjugating Enzyme E2” (Supporting Information S1: Table 1) [25]. During data integration, any samples with missing survival time or status information were excluded from the analysis.

### 2.2 Constructing UBE2‐Associated Subtypes

Univariate analysis using UBE2‐related genes with a screening criterion of *p* < 0.05 was utilized to identify genes associated with HCC prognosis. Sample stratification was derived via the “ConsensusClusterPlus” algorithm in R, which generated consensus partitions by performing ten rounds of resampling. The final cluster count was selected by examining the cumulative distribution function (CDF) curves together with the mean stability of each cluster. Additionally, differentially expressed genes (DEGs) between the subtypes were identified using the R package “limma,” followed by Gene Ontology (GO) and Kyoto Encyclopedia of Genes and Genomes (KEGG) analyses to investigate the associated biological pathways.

### 2.3 Risk Model Establishment

To identify prognostic genes for model construction, univariate Cox regression was performed on 1978 subtype‐specific DEGs, yielding 158 genes significantly associated with overall survival (*p* < 0.01). These candidate genes were then used to develop a prognostic risk model via LASSO regression. Patient risk scores were calculated as: RiskScore=∑Coef(i)*Exp(i). Samples were stratified into low‐ and high‐risk groups according to whether their risk score was above or below the median value.

### 2.4 Construction and Validation of Prognostic Nomograms

Cox regression analyses were performed to test the independence of the risk score as a prognostic factor. Time‐dependent ROC curves assessed its predictive performance at 1, 3, and 5 years. A nomogram integrating the risk score with multivariate‐selected clinical variables was constructed, with calibration curves and decision curve analysis (DCA) evaluating its predictive accuracy and clinical utility.

### 2.5 Immune Cell Infiltration Analysis

To assess immune characteristics across risk groups, CIBERSORT was first used to estimate the relative abundances of immune cell types. Subsequently, the ESTIMATE algorithm was then applied to calculate stromal, immune, and ESTIMATE scores, as well as tumor purity, with violin plots used to visualize differences between groups. In addition, Single‐sample gene set enrichment analysis (ssGSEA) was performed to quantify immune cell infiltration and immune‐related functional pathways. ICP expression was compared between groups using boxplots. Tumor samples were additionally classified into six immune subtypes based on previously published data [26]. Finally, potential immunotherapy responses for individual patients were predicted using the Tumor Immune Dysfunction and Exclusion (TIDE) algorithm.

### 2.6 Enrichment Analysis

The DEGs were identified by using “limma” package in R software between the high‐ and low‐risk groups, with selection criteria of |log FC | > 1 and *p* < 0.05, and GO analysis and KEGG pathway were conducted to elucidate the potential mechanisms via the “clusterProfiler” package. The samples were divided into high‐ and low‐risk groups based on the median risk score, and Gene Set Enrichment Analysis (GSEA) was used for pathway enrichment in each group.

### 2.7 Drug Sensitivity Prediction

We performed screening of targeted and anti‐tumor drugs significantly associated with UBE2‐related genes sensitivity and prognosis using the CellMiner database. Furthermore, we applied the DGIdb database to identify drugs that interact with signature genes and discuss potential drug targets for disease genes and the direction of action. The predicted half‐maximal inhibitory concentration (IC50) values for multiple therapeutic agents were obtained using the “pRRophetic” package.

### 2.8 Cell Lines and Quantitative Reverse Transcription‐PCR(qRT‐PCR)

THLE‐2 normal hepatocytes and Huh7 liver cancer cells (Chinese Academy of Sciences, Shanghai, China) were cultured in DMEM supplemented with 10% FBS and 10,000 U/mL penicillin–streptomycin at 37°C with 5% CO₂. Total RNA was extracted using the RNA Eazy Fast Cell Kit and reverse‐transcribed into cDNA with the FastKing RT Kit. Quantitative real‐time PCR was performed on a StepOnePlus system using SuperReal PreMix Plus (TIANGEN Biotech, Beijing), and relative expression was determined by the 2^−△△CT^ method (primer sequences in Table 1).

**Table 1:** Sequences of primers for qRT‐PCR.

Gene name	Primer sequences (5′‐3′)
FTCD	Forward: CAGAGCTGTGACCTCGAAAC
	Reverse: GTACCGTGCTCTGTGGAACT
LGALS3	Forward: GTCCGGAGCCAGCCAAC
	Reverse: ACGCATCATGGAGCGAAAAA
PAGE1	Forward: CATCTGGTAGATCCGCAGGC
	Reverse: AAAACCCATATTTCACAACTTCAGC
RAMP1	Forward: CTGAGAAATCCGGCCCATCA
	Reverse: CACCGTAGTTAGCCTCCTGG
RAMP3	Forward: CTGCTCTGCGGTGGGTG
	Reverse: CCACCTTGCCCATCATGTCT
SLC1A7	Forward: CTGCCTCCTCGGCTTCTTC
	Reverse: TCCGGACATCAAGCTGGAGA

## 3 Results

### 3.1 Construction of UBE2‐Related Molecular Subtypes Based on Prognosis‐Related Genes

**Figure 1:**
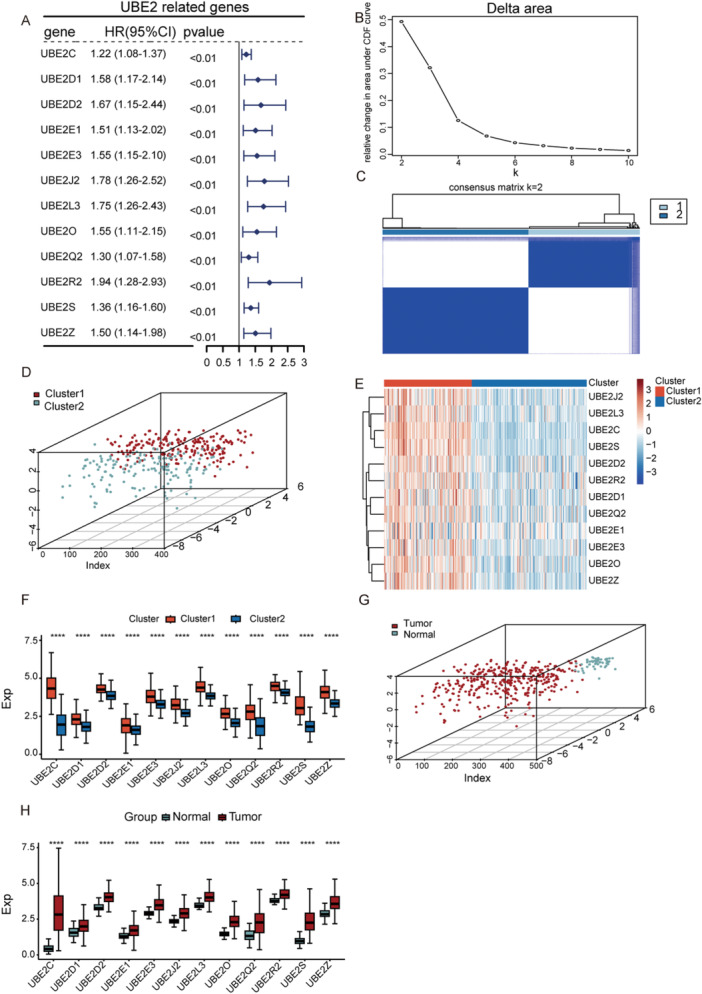
UBE2‐related molecular subtypes in liver cancer. (A) Forest plot showing UBE2‐related genes significantly associated with prognosis. (B) Relative increase in area under the CDF curve with different numbers of assumed subtypes. (C) Consensus clustering matrix indicating two molecular subtypes in TCGA‐LIHC. (D) Principal component analysis (PCA) of the two subtypes. (E) Heatmap of UBE2 prognosis‐related gene expression across subtypes. (F) Boxplot comparing expression of UBE2 prognosis‐related genes between subtypes. (G) PCA of LIHC and normal samples. (H) Boxplot of UBE2 gene expression in tumor versus normal tissues. ****: *p* < 0.0001.

In our study, univariate Cox regression identified 12 UBE2‐related genes significantly associated with prognosis (Figure 1A). Consensus clustering based on the CDF indicated that the optimal separation was achieved when two subtypes were assumed (Figure 1B,C). Principal Component Analysis (PCA) further validated the distinction between these two subtypes (Figure 1D). To investigate subtype differences, we plotted a heatmap and boxplots of the 12‐gene expression profiles (Figure 1E,F). Moreover, PCA of both normal and tumor samples demonstrated a clear separation along the principal components (Figure 1G), and the expression of all 12 UBE2‐related genes was significantly upregulated in tumor tissues compared with normal tissues (Figure 1H).

### 3.2 Identification of Prognostic Biomarkers Based on HCC Molecular Subtypes

To evaluate survival differences between the two subtypes, Kaplan–Meier analysis revealed that patients in Cluster 1 exhibited significantly poorer prognosis compared with Cluster 2 (Figure 2A). Differential expression analysis identified 1978 overlapping genes between the subtypes (adjusted *p* < 0.01, |logFC | > 0.585; Figure 2B). KEGG pathway enrichment analysis was conducted on these DEGs. Upregulated genes were primarily enriched in complement and coagulation cascades, drug metabolism–cytochrome P450, and xenobiotic metabolism (Figure 2C), while downregulated genes were associated with similar metabolic and signaling pathways (Figure 2D). These findings suggest that metabolic processes and immune‐related pathways may contribute to the molecular differences between HCC subtypes.

Figure 2Identification of prognostic UBE2‐related genes in HCC. (A) Kaplan–Meier survival analysis of patients stratified by molecular subtypes. (B) Volcano plot showing differentially expressed genes (DEGs) between the two molecular subtypes. (C, D) KEGG pathway enrichment analyses of upregulated (C) and downregulated (D) DEGs. (E, F) GO enrichment analyses of upregulated (E) and downregulated (F) DEGs. (G) LASSO coefficient profiles of prognostic genes. (H) Confidence intervals under lambda. (I) Forest plot of multivariate Cox regression analysis for overall survival in HCC patients. (J) Expression levels of the signature genes FTCD, LGALS3, PAGE1, RAMP1, RAMP3, and SLC1A7. ns: not significant, ***: *p* < 0.001, ****: *p* < 0.0001, GO, gene ontology; KEGG, Kyoto Encyclopedia of Genes and Genomes; LASSO, least absolute shrinkage and selection operator.

**Figure:**
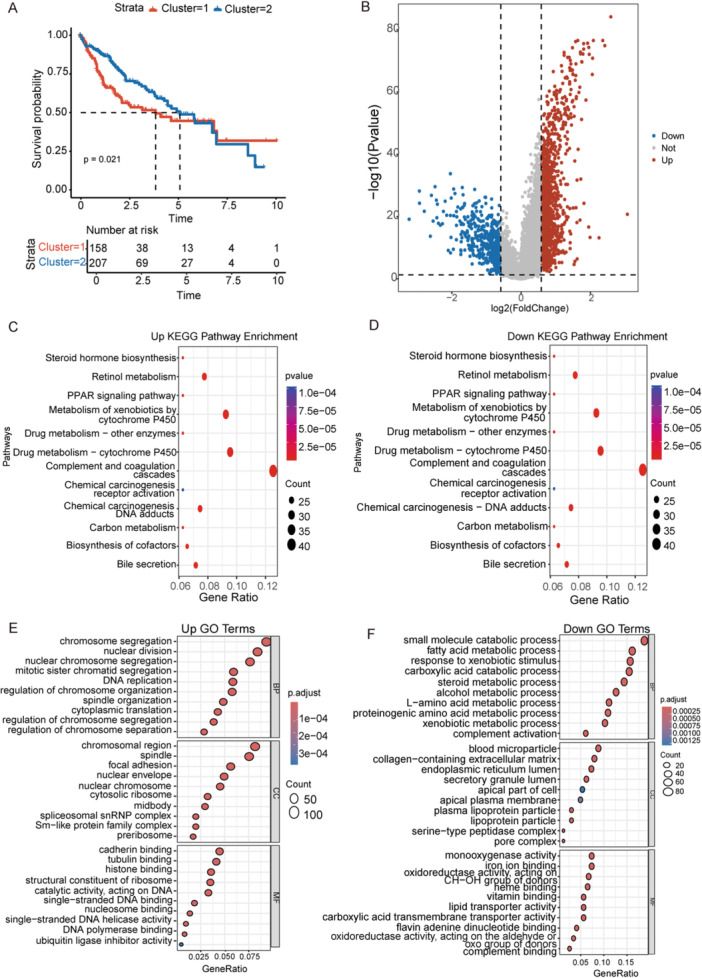

**Figure:**
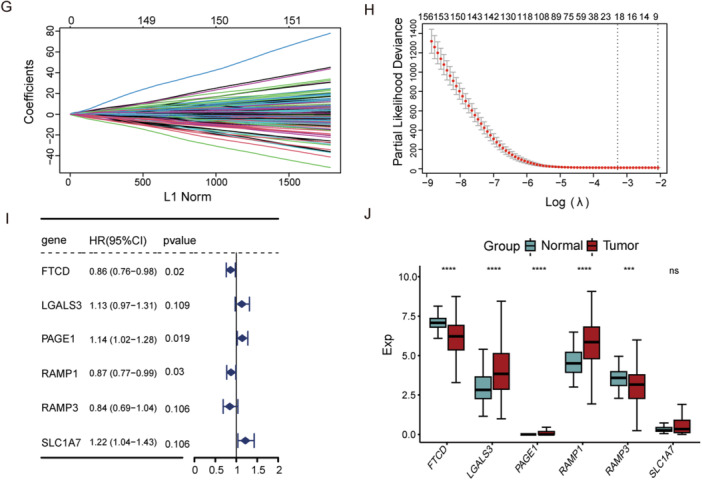

GO analysis indicated that upregulated genes were mainly involved in chromosome segregation (biological process), chromosomal region (cellular component), and cadherin binding (molecular function), whereas downregulated genes were enriched for small molecule catabolic processes, blood microparticle, and monooxygenase activity (Figure 2E,F). Subsequently, candidate prognostic genes were identified using univariate Cox analysis, LASSO, and stepwise regression (Figure 2G,H). Stepwise multivariate Cox regression further refined the model, resulting in six key prognostic genes, with the corresponding risk score calculated as follows (Figure 2I).

Riskscore=0.1227*LGALS3−0.1491*FTCD+0.1331*PAGE1−1352*PAMP1−0.1695*PAMP3+0.1960*SLC1A7

In addition, we found that the gene expression levels of FTCD, LGALS3, PAGE1, RAMP1, and RAMP3 were significantly different in tumor samples relative to normal samples, whereas SLC1A7 showed no significant difference (Figure 2J).

### 3.3 Validation of UBE2‐Related Prognostic Models

**Figure 3:**
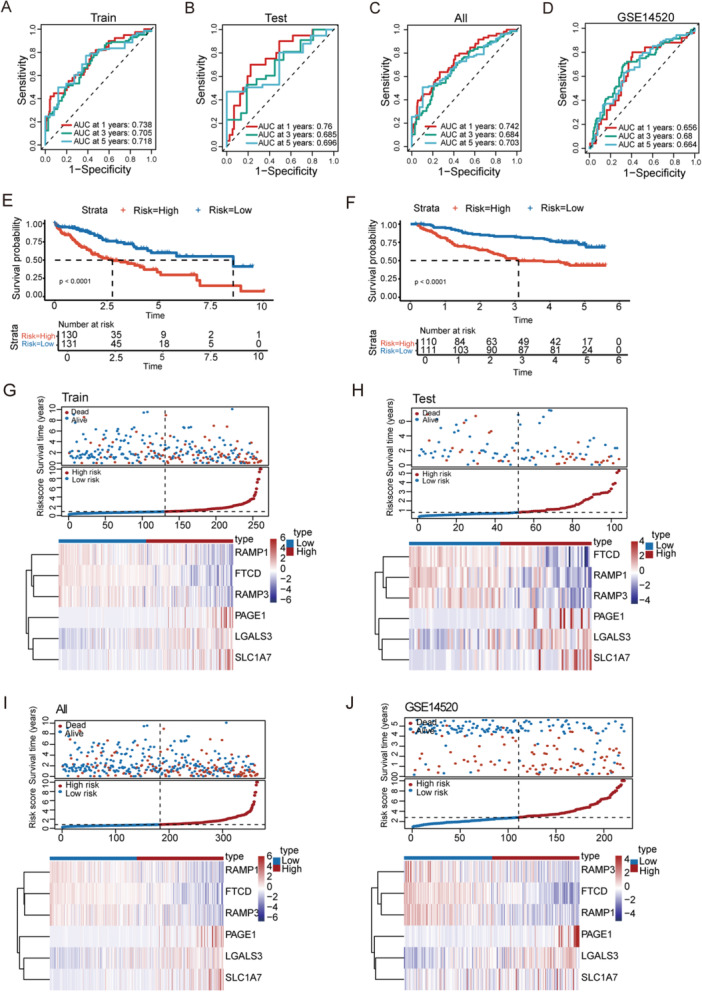
Prognostic performance of the UBE2‐related gene signature in HCC. (A–D) Time‐dependent ROC curves for 1−, 3−, and 5‐year overall survival in the TCGA training set (A), TCGA testing set (B), TCGA entire cohort (C), and the GSE14520 validation cohort (D). (E, F) Kaplan–Meier survival analyses comparing the high‐ and low‐risk groups in the TCGA training cohort (E) and the GSE14520 cohort (F). (G–J) Distribution of risk scores and survival status of patients in the TCGA training cohort (G), TCGA testing cohort (H), TCGA entire cohort (I), and the GSE14520 cohort (J). ROC, Receiver operating characteristic, TCGA, The Cancer Genome Atlas.

To evaluate model performance, patients in TCGA and GEO cohorts were assigned risk scores and divided at the median into high‐ and low‐risk groups. Time‐dependent ROC curves yielded AUCs > 0.65 for 1−, 3−, and 5‐year OS in all cohorts, demonstrating reliable prognostic accuracy (Figure 3A–D). Kaplan–Meier curves demonstrated markedly longer survival in the low‐risk group relative to the high‐risk group (Figure 3E,F). Furthermore, increasing risk scores were associated with significantly worse survival outcomes and shorter life expectancy (Figure 3G–J).

### 3.4 Construction of Nomogram for Independent Prognostic Analyses in HCC Patients

**Figure 4:**
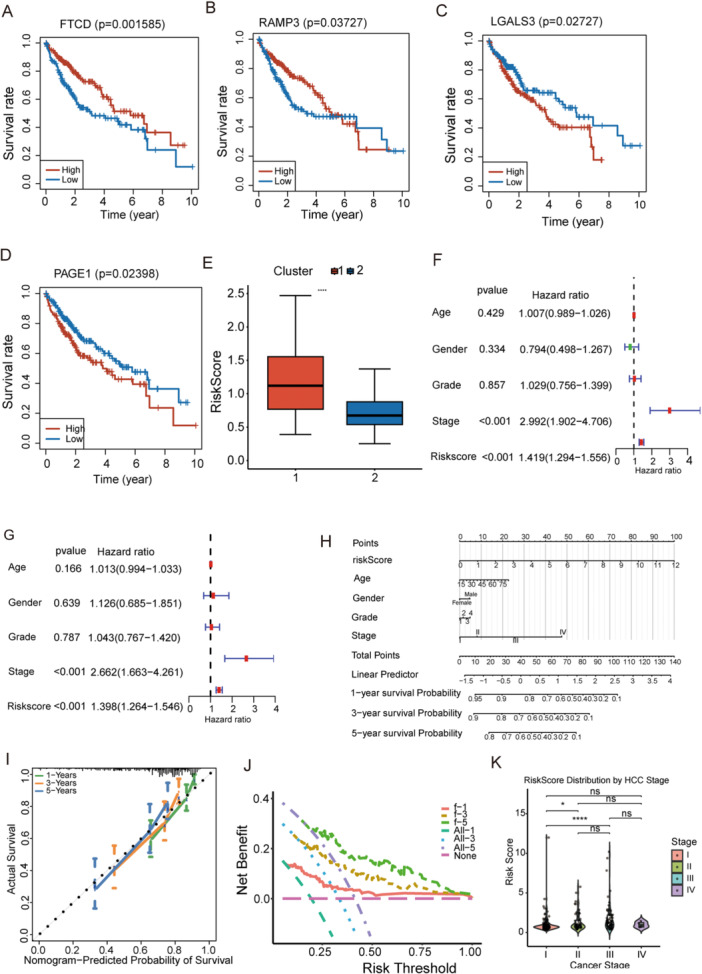
Identification of independent prognostic factors and construction of a prognostic nomogram. (A–D) Kaplan–Meier survival curves of the signature genes FTCD, RAMP3, LGALS3, and PAGE1. (E) Distribution of risk scores across molecular clusters. (F, G) Univariate (F) and multivariate (G) Cox regression analyses evaluating the prognostic value of the risk score and clinical variables. (H) Nomogram integrating the risk score with clinical variables for survival prediction in patients with HCC. (I, J) Calibration curve (I) and decision curve analysis (DCA) (J) assessing the performance of the nomogram. (K) Comparison of risk scores across clinical stages. ns: not significant, *: *p* < 0.05, ****: *p* < 0.0001.

High FTCD and LGALS3 expression was associated with better survival, whereas high RAMP3 and PAGE1 expression was associated with worse survival. RAMP1 showed no significant effect (Figure 4A–D), consistent with the constructed risk score formula. Risk scores were compared between the two molecular subtypes, revealing that Cluster 1 exhibited higher scores than Cluster 2 (Figure 4E). Univariate and multivariate Cox regression analyses identified stage and risk score as key prognostic factors independent of other clinical variables (Figure 4F,G). A nomogram integrating risk score and stage was then constructed to estimate patient‐specific survival probabilities (Figure 4H). Calibration curves showed close alignment between predicted and observed 1−, 3−, and 5‐year survival rates, indicating excellent predictive accuracy (Figure 4I). DCA further demonstrated the clinical benefit of the nomogram compared with extreme scenarios (Figure 4J). Risk scores differed significantly between Stage I and Stage II and between Stage I and Stage III, with a general upward trend across stages. These findings suggest that early‐stage disease harbors molecular characteristics distinct from those of intermediate stages (Figure 4K).

### 3.5 Immune Infiltration Analysis of UBE2‐Related Signatures and Prediction of Immunotherapy

To characterize the immune cell infiltration landscape across risk groups, we generated comprehensive heatmaps and box plots (Figure 5A,B), illustrating the distribution of various immune cells. To further estimate immune infiltration, we calculated immune, tumor purity, stromal, and ESTIMATE scores (Figure 5C). Notably, the low‐risk group exhibited a higher stromal score, indicating increased stromal cell infiltration, while immune, tumor purity, and ESTIMATE scores showed no significant differences. Heatmaps and boxplots of 28 immune‐related cells and functions (Figure 5D,E) revealed that nearly half displayed significant differences between risk groups. To evaluate potential immunotherapy responses, we compared TIDE, ICP, and immunophenoscore (IPS) scores. The low‐risk group demonstrated a favorable immunoprofile, characterized by lower TIDE/ICP and higher IPS (Figure 5F–H), which implies impaired immune evasion and a more promising immunotherapy outlook. Additionally, the two risk groups corresponded to distinct immune subtypes(Figure 5I), highlighting heterogeneity in the tumor immune microenvironment.

Figure 5Immune landscape differences between the high‐ and low‐risk groups. (A) Relative distribution of immune cell fractions in the high‐risk and low‐risk groups. (B) Comparison of immune cell infiltration levels between the two risk groups. (C) Comparison of tumor purity, immune score, stromal score, and ESTIMATE score between the high‐risk and low‐risk groups. (D) Heatmap showing immune cell infiltration and immune‐related functional signatures in the two risk groups. (E) ssGSEA scores of immune checkpoint–related gene sets in the high‐risk and low‐risk groups. (F) TIDE score comparison between the high‐risk and low‐risk groups. (G) Expression levels of immune checkpoint genes in the two risk groups. (H) Comparison of immunophenoscore (IPS) between the high‐risk and low‐risk groups. (I) Alluvial diagram illustrating the relationships among molecular clusters, risk groups, and immune subtypes. *: *p* < 0.05, **: *p* < 0.01, ***: *p* < 0.001, ****: *p* < 0.0001; ns, not significant, ssGSEA, single sample Gene Set Enrichment Analysis, TIDE, tumor immune dysfunction and exclusion.

**Figure:**
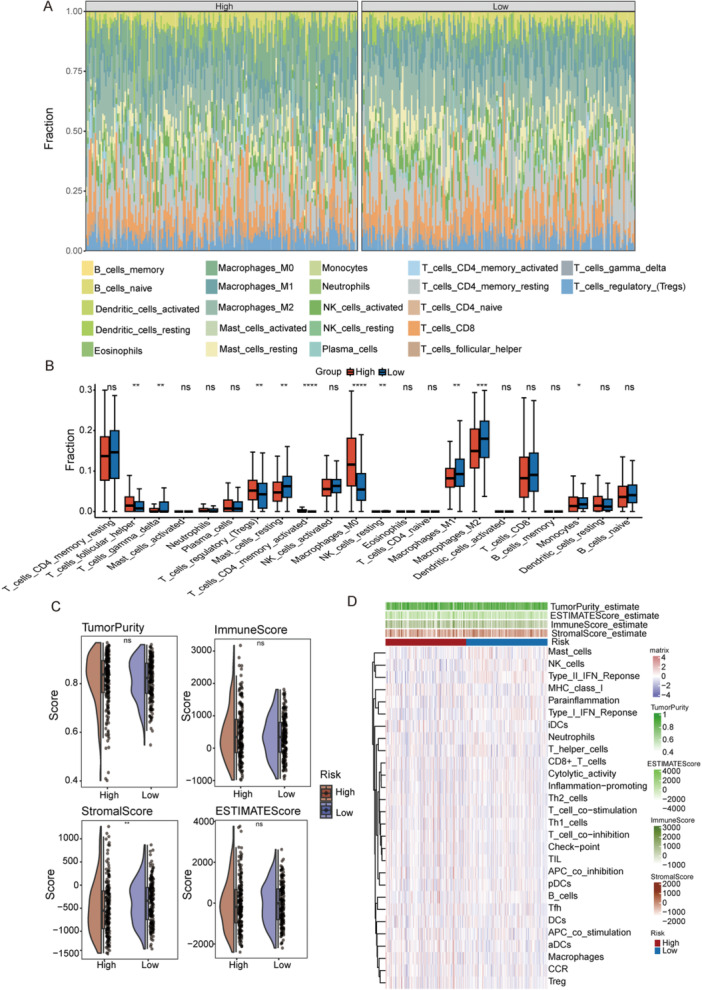

**Figure:**
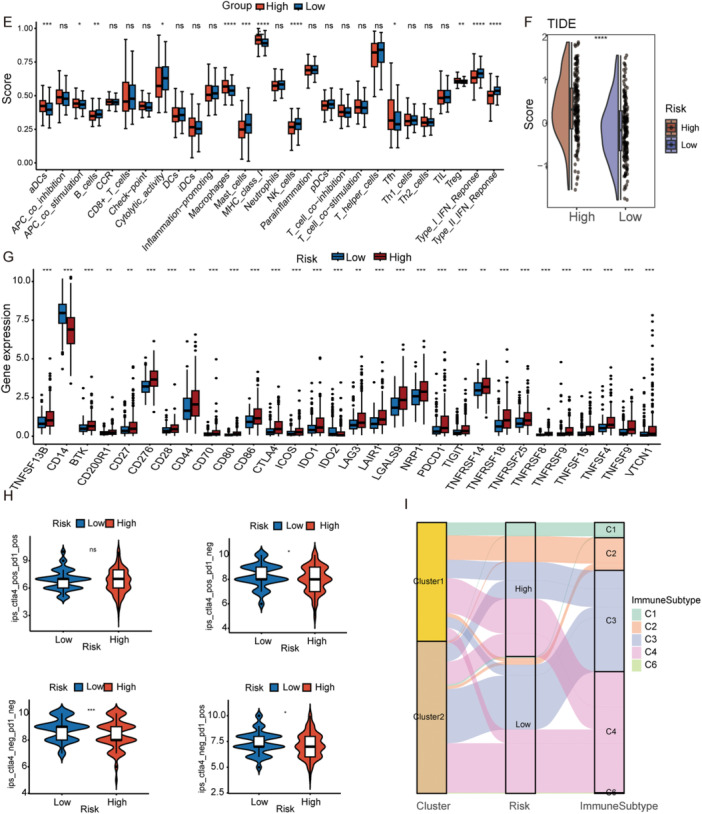

### 3.6 Enrichment Analysis Across Risk Groups

**Figure 6:**
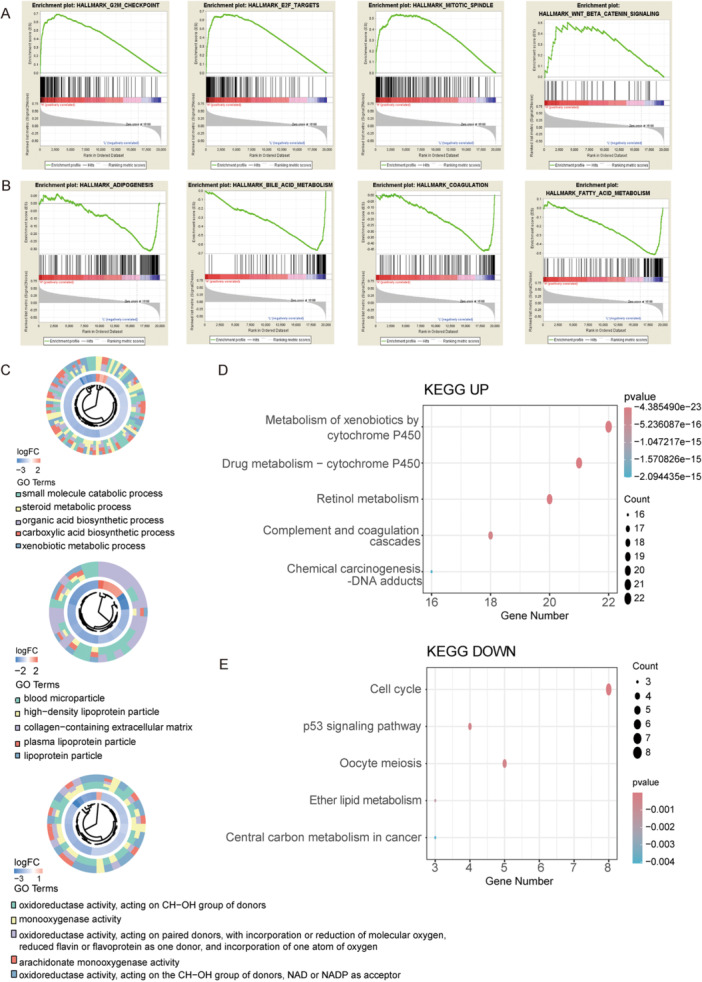
Functional enrichment analyses between the high‐ and low‐risk groups. (A, B) GSEA of Hallmark pathways in the high‐risk (A) and low‐risk (B) groups. (C) GO enrichment analysis of differentially expressed genes between the two risk groups. (D,E) KEGG pathway enrichment analyses of upregulated (D) and downregulated (E) genes between the high‐ and low‐risk groups. GSEA, Gene set enrichment analysis, GO, Gene Ontology, KEGG, Kyoto Encyclopedia of Genes and Genomes.

Differential expression analysis between risk groups identified 230 DEGs, including 63 upregulated and 167 downregulated genes. GSEA revealed enrichment of G2M checkpoint, E2F targets, and mitotic spindle pathways in the high‐risk group (Figure 6A), and adipogenesis, bile acid metabolism, and coagulation pathways in the low‐risk group (Figure 6B). GO analysis indicated that DEGs were involved in steroid metabolic processes. KEGG analysis showed that upregulated DEGs were enriched in xenobiotic metabolism by cytochrome P450, drug metabolism— cytochrome P450, retinol metabolism, and complement and coagulation cascades (Figure 6D), while downregulated DEGs were associated with cell cycle, oocyte meiosis, and p53 signaling pathways (Figure 6E).

### 3.7 Drug Sensitivity Analysis in Patients With HCC

**Figure 7:**
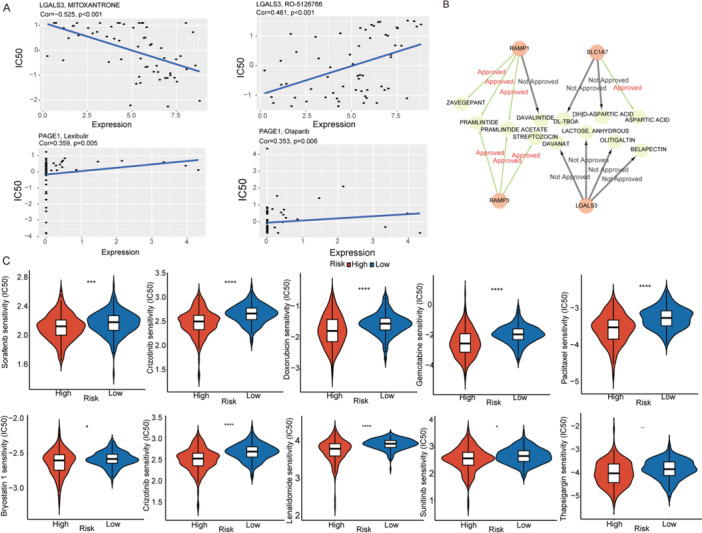
Drug sensitivity analysis based on the risk model. (A) Correlations between the expression levels of model genes and drug sensitivity across multiple compounds using the CellMiner database. (B) Predicted drug–gene interactions involving the signature genes LGALS3, PAGE1, RAMP1, and SLC1A7. (C) Estimated IC50 values of 5‐Fluorouracil and Gemcitabine in the high‐ and low‐risk HCC groups. *: *p* < 0.05, ***: *p* < 0.001, ****: *p* < 0.0001.

CellMiner analysis showed that LGALS3 expression negatively correlated with Mitoxantrone (Cor = −0.525, *p* < 0.001) and positively with RO‐5126766 (Cor = 0.461, *p* < 0.001), while PAGE1 expression positively correlated with Lexibulin (Cor = 0.359, *p* < 0.001) and Olaparib (Cor = 0.353, *p* < 0.001) (Figure 7A). DGIdb query identified additional drugs interacting with signature genes (Figure 7B). IC50‐based predictions indicated consistently lower IC50 values in the high‐risk group across Sorafenib, Crizotinib, Doxorubicin, Gemcitabine, Paclitaxel, Bryostatin 1, Crizotinsib, Lenalidomide, Sunitinib, and Thapsigargin suggesting greater sensitivity to these agents in high‐risk patients (Figure 7C, Supporting Information S1: Table 2).

### 3.8 Expression of the Feature Genes Comprising the Risk Model Was Validated by qRT‐PCR

**Figure 8:**
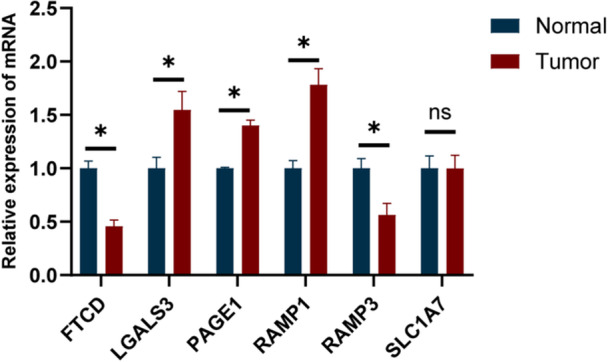
Validation of characteristic gene expression by qRT‐PCR. The relative mRNA expression levels of FTCD, LGALS3, PAGE1, RAMP1, RAMP3, and SLC1A7 were measured by qRT‐PCR. Data are presented as mean ± SD. *p* < 0.05; ns, not significant.

To independently confirm the expression of the candidate signature genes, we conducted qRT‐PCR assays. In contrast to the marked downregulation of FTCD and RAMP3, tumor tissues exhibited elevated expression of LGALS3, PAGE1, and RAMP1 compared to normal samples (Figure 8). These findings suggest that dysregulation of these genes may contribute to HCC malignancy. No differential expression was observed for SLC1A7. Overall, the qRT‐PCR results were consistent with our transcriptomic data.

## 4 Discussion

In this study, we identified two distinct HCC subtypes based on UBE2‐related genes and developed a prognostic model comprising FTCD, LGALS3, PAGE1, RAMP1, RAMP3, and SLC1A7. This model demonstrated robust predictive, immunological, and pharmacological utility, effectively segregating patients into high‐ and low‐risk categories with divergent survival. Importantly, these findings were validated across multiple cohorts, and potential therapeutics were suggested via drug sensitivity analysis, providing insights for personalized HCC management.

Existing evidence suggests that six genes influence various tumor processes via diverse mechanisms. A study by Wang et al. demonstrated that the lower expression levels of FTCD are significantly downregulated in HCC, which was consistent with our results [27]. Loss of FTCD in the liver leads to lipid buildup and hepatocarcinogenesis through PPAR gamma and SREBP2 [27]. These results suggest that FTCD expression modulates HCC risk and may have predictive value for disease onset. LGALS3 (galectin‐3) mediates tumor immunosuppression and cellular regulation, is upregulated in various cancers, and predicts poor HCC prognosis [28]. LGALS3 was a risk factor in our study, aligning with Dong et al., who reported it as a biomarker for progression from chronic HBV infection to HCC [29]. PAGE1 is a member of a gene family typically expressed in tumors while largely absent from normal tissues, with the exception of the testis [30]. PAGE1 may contribute to cancer progression, but its molecular mechanisms remain largely unclear. Li [31] and Cui [32] developed a signature, including PAGE1, to predict HCC prognosis. In our study, RAMP1 and RAMP3 showed opposite expression patterns in cancer and non‐cancer tissues: RAMP1 was upregulated while RAMP3 was downregulated in HCC. Existing studies on their roles in HCC are limited. RAMP1 has been reported to promote tumorigenesis in prostate cancer [33], whereas RAMP3 has been identified as a key gene in several prognostic models [34, 35, 36]. This contrasting expression suggests that these two RAMP family members may play distinct roles in HCC progression. RAMP1 upregulation could contribute to tumor‐promoting signaling pathways [37], potentially enhancing cell proliferation or survival, while RAMP3 downregulation may impair protective or anti‐tumor mechanisms [38]. SLC1A7 showed no significant expression change between tumor and normal tissues in our study, yet it is reported as a prognostic marker in non‐small cell lung cancer [39] and HCC [39], suggesting a potential subtle role in prognosis that warrants further investigation. In conclusion, our results and prior research collectively demonstrate the critical role of these six UBE2‐related genes in HCC, thus nominating them as promising targets for future therapy.

The pivotal impact of the tumor microenvironment on tumor phenotypes is well established. To clarify the involvement of immune cells in HCC progression, we assessed immune‐related functional pathways and infiltration levels of multiple immune cell types. Significantly increased T cell infiltration was observed in the low‐risk group. It is well established that T cells, central mediators of adaptive immunity, are crucial for antitumor immunity, and T‐cell depletion markedly influences patient prognosis in HCC [40]. As a central feature of the tumor microenvironment, immune cell infiltration contributes to tumor immune escape and the establishment of an inflammatory milieu [41]. Accordingly, we further examined infiltration differences between risk groups. Prior research has shown that higher levels of naïve and memory B cells correlate with better survival in HCC patients [42], consistent with our observations. ICIs have become a central therapy for advanced, unresectable HCC [43]. Analysis of ICP gene expression revealed elevated levels in the low‐risk group compared to the higher‐risk group. Because tumor‐cell‐associated ICPs facilitate immune evasion [44], these results suggest that low‐risk patients may be less prone to immune escape during immunotherapy. Moreover, the low‐risk group showed higher IPS and lower TIDE scores, indicating stronger immunogenicity and greater predicted sensitivity to immunotherapy [45].

Although our study provides clinically relevant insights for prognostic assessment in HCC, several limitations remain. First, the lack of uniform clinical data, including HCC etiology and prior treatments, limited their inclusion in our analyses and warrants cautious interpretation. Second, our findings are based on in silico analyses of bulk transcriptomic data, which may be influenced by cohort‐specific biases or batch effects. While emerging approaches such as single‐cell or spatial transcriptomics can capture cellular heterogeneity and spatial context, they remain limited by sample size, cost, and analytical complexity. Therefore, validation of our risk signature in larger, independent cohorts is necessary, and complementary single‐cell or spatial analyses could further refine the mechanistic understanding of UBE2‐associated genes. Finally, experimental studies are required to elucidate their immune‐related functions and guide potential therapeutic applications.

## 5 Conclusion

In summary, our research formulated a prognostic risk model for HCC, demonstrating robust predictive performance. We explored the correlations groups stratified by risk scores and various factors, including immune cell infiltration, responses to immunotherapy, as well as drug sensitivity profiles. The results of our study provided new perspectives on survival prognosis and clinical manifestations with HCC patients.

## Author Contributions

Y.T., C.W.L., X.D.M., L.Y.K., W.L., and S.Q.Z. contributed to the study design. Y.T. and C.W.L. conducted the literature search. X.D.M. and L.Y.K. acquired the data. W.L. wrote the article. S.Q.Z. performed data analysis. S.Q.Z. revised the article and gave the final approval of the version to be submitted. All authors read and approved the final manuscript.

## Funding

The authors received no specific funding for this work.

## Ethics Statement

The authors have nothing to report.

## Consent

The authors have nothing to report.

## Conflicts of Interest

The authors declare no conflicts of interest.

## Supporting information

Supplementary table.

## Data Availability

The data and materials in the current study are available from the corresponding author on reasonable request.
